
JOURNAL INFORMATION
==============================
NLM Title Abbreviation: Buffalo Med J Mon Rev Med Surg Sci
NLM Journal ID: 0004276

The Buffalo Medical Journal and Monthly Review of Medical and Surgical Science

Publisher: Biomedical Journal Digitization Project

ARTICLE INFORMATION
==============================
PMCID: PMC8932446
PMID: 35376943
Article version: 1
Subjects: Editorial Department

Meeting of the Medical Society of South-Western New York

Print publication date: 1858 Dec
Volume: 14
Issue: 7
First page: 443
Last page: 445
License: This work is free of known copyright restrictions. For more information, please see PMC Back Issue Digitization.
License URL: https://creativecommons.org/publicdomain/mark/1.0/

==============================

of the practitioner, but must be a great relief to friends who otherwise might not have believed that all had been done.

It is only a few days ago that a patient from whom we had removed the matrix of the nail of the great toe, for onychia maligna, after the nail had previously been removed by the ordinary operation, and after she had suffered for three years, told us that a homœopathic practitioner volunteered the valuable information, that now her toe was healed up she would die of consumption. This *professional gentleman* was working with her husband in a blacksmith's shop, only a short time before, and from thence proceeded to practice medicine, when he had promised to cure her toe himself with the inevitable sugar of milk. To be classed with such animals is humiliating, but it is one of the crosses which we knew we should have to bear when we took up our profession, and we suppose we have no right to complain.

---

*Meeting of the Medical Society of South-Western New York.*—Every half year we receive an account of a meeting of this society, and judge from what we see that it is always a pleasant affair. When doctors feel a disposition to come together as brethren, they are the most companionable of men; good feeling alone prevails, and this appears always to be the case with our brethren of South-Western New York.

The society met on the first Wednesday in November, and elected the following gentlemen officers for the ensuing year:

*President*, H. M. T. Smith, Dunkirk.
*Vice-President*, Edson Boyd, Ashville.
*Secretary and Treasurer*, Charles K. Irwin, Dunkirk.
*Executive Committee*, Dr. G. C. Bennet, Quincy,
" A. K. Avery, Forestville,
" G. S. Harrison, Sinclearville.
*Com. of Arrangements for* 1859, Dr. Boyd, Ashville,
Dr. Thomas, Dewittville,
" Fenwick, Mayville.

The following resolutions, among others, were unanimously adopted:

*Resolved*, That the members of the Medical Society of South-Western New York will not, in any instance, visit, examine or prescribe for a patient of an irregular practitioner of medicine, until the patient, or his or her guardian, shall have assured us of the discharge of the irregular practitioner, and our employment.

*Resolved*, That the Medical Society of South-Western New York endorse, as professional, the administration of chloroform in obstetrics; and we assure the public that its use requires the same, and no more skill than that of other potent remedies; and that we recommend, as safe administrators of it, any and all regular members of our society and profession as being equally competent, who desire to assume the responsibility of its administration.

After the meeting, the members, with their wives and invited guests, sat down to an excellent dinner, which was enlivened by good speeches by the president, Dr. Smith, and Dr. Washburn, as well as some who were not members of our profession. We are sorry to be only able to speak in terms of general commendation of these responses, which are published in full in the "Dunkirk Press and Western Argus."

We like the spirit of the first resolution: too often is the practitioner neglectful of inquiring, when called, if any other practitioner, and especially an irregular, is in attendance. We are thus subjected to the unpleasant chance of meeting a quack at the house of our patient, and often under the disagreeable necessity of controling our desire to forcibly eject him.

We do not know what some of our Philadelphia, Boston, or New York friends would say to the second resolution. It is now becoming quite common in these cities, to repudiate chloroform and use nothing but ether, especially in the hospitals and clinics. The horror of the eminent Professor of Obstetrics in the Jefferson School, when he barely hears of the contemplated administration of chloroform, could not be greater than if he were asked to witness a wilful murder; indeed it is a tradition that he was accustomed to kill sheep with chloroform before the class, to inspire in them a measure of detestation of the article. This feeling is very common in Philadelphia, which we look to rather as the fountain head of medicine in this country. True it is, that a physician or surgeon, if he administers chloroform frequently, will sometimes see a case, where the escape from death will be *very narrow*. We have seen one or two such instances in the practice of eminent surgeons, and though we much prefer this form of anæsthetic from its pleasant odor, its rapidity and certainty of action, and do administer it, we are always rejoiced when the patient is well out of danger from its effects. The total arrest of respiration which sometimes occurs in its administration, and which frequently continues long enough to excite the most lively apprehension, is a fearful thing for an operator, and an experience which we hope never to undergo. For all that, however, chloroform is so much more agreeable, rapid, and certain in its action than ether, and the cases of death from its administration, with proper precaution, are so rare, that we always give it the preference. We regard ether, however, as absolutely without danger. What the anæsthetic *acétone*, which is described in Brown-Séquard's Journal and which we referred to in our last number, may prove to be, we do not know; we hope that some agent may be discovered which will prove as effectual as chloroform, and as safe as ether.

The society adjourned, Nov. 4th, to meet at Mayville on the second Wednesday in May, 1859.

---

*Letter detailing a case of Criminal Abortion and Infanticide.*—We publish the following letter, which appears to have been called out by our article on *Criminal Abortions*, which details one of the most horrible instances of depravity, is rather insanity, which we have ever known. This is one of the effects of advertising abortion pills; and that such a thing should ever occur in a civilized land would be a matter of astonishment to any but the physician, who sees so much misery and crime. Cannot this matter be remedied, and will not our brother editors aid us in the effort to awaken public sentiment in this direction?

Dr. Flint—Dear Sir: I see by the two last numbers of the Buffalo Medical Journal that you have undertaken to correct and change the puhlic mind, and so do away with a great moral evil (criminal abortions). I am glad, for, one that the medical faculty are getting aroused to this great subject, and that they may show its true character and influence, and correct, if possible, the error. It is a great evil, and there must be measures and means advanced to check it, or where will it end?

I will give you the history of a case that occurred to me in my practice only a year ago.

Mrs. W., aet forty, widow woman, with three children, applied at my office for medical aid; said she had been unfortunate, and got caught in a bad scrape, and wished something to produce the desired effect. She was told that it was very dangerous to give such medicine, and that they were very uncertain in their effects, as no specific medicine was known that would produce this effect without danger. She left the office, and nothing more was heard from her for about two weeks, when I was summoned to her house as soon as I could get there. I was some twelve hours before I arrived, and when I did, I learned a tale that makes my blood almost chill
